# Single cell analysis of transcriptome and open chromatin reveals the dynamics of hair follicle stem cell aging

**DOI:** 10.3389/fragi.2023.1192149

**Published:** 2023-07-03

**Authors:** Chi Zhang, Dongmei Wang, Robin Dowell, Rui Yi

**Affiliations:** ^1^ Department of Pathology, Northwestern University Feinberg School of Medicine, Chicago, IL, United States; ^2^ Department of Dermatology, Northwestern University Feinberg School of Medicine, Chicago, IL, United States; ^3^ Robert H. Lurie Comprehensive Cancer Center, Northwestern University Feinberg School of Medicine, Chicago, IL, United States; ^4^ BioFrontiers Institute, University of Colorado Boulder, Boulder, CO, United States

**Keywords:** hair follicle stem cell, stem cell exhaustion, quiescence, cell adhesion, single cell genomics, scRNAseq, scATACseq

## Abstract

Aging is defined as the functional decline of tissues and organisms, leading to many human conditions, such as cancer, neurodegenerative diseases, and hair loss. Although stem cell exhaustion is widely recognized as a hallmark of aging, our understanding of cell state changes–specifically, the dynamics of the transcriptome and open chromatin landscape, and their relationship with aging–remains incomplete. Here we present a longitudinal, single-cell atlas of the transcriptome and open chromatin landscape for epithelia cells of the skin across various hair cycle stages and ages in mice. Our findings reveal fluctuating hair follicle stem cell (HF-SC) states, some of which are associated with the progression of the hair cycle during aging. Conversely, inner bulge niche cells display a more linear progression, seemingly less affected by the hair cycle. Further analysis of the open chromatin landscape, determined by single-cell Assay for Transposase-Accessible Chromatin (ATAC) sequencing, demonstrates that reduced open chromatin regions in HF-SCs are associated with differentiation, whereas gained open chromatin regions in HF-SCs are linked to the transcriptional control of quiescence. These findings enhance our understanding of the transcriptional dynamics in HF-SC aging and lay the molecular groundwork for investigating and potentially reversing the aging process in future experimental studies.

## Introduction

Aging is an inevitable process characterized by the gradual accumulation of damages in cells and tissues, leading to declines in tissue functions and increased vulnerability to chronic diseases and mortality (Holliday, 1995; Campisi et al., 2019; López-Otín et al., 2023). Although significant progress has been made in understanding the mechanisms of aging (Van Deursen, 2014; Revuelta and Matheu, 2017), many questions regarding molecular and cellular changes and cell-to-cell variation during aging remain unanswered (Martinez-Jimenez et al., 2017; Todhunter et al., 2018).

As the largest organ in mammals including human, the skin and its appendages provide an excellent experimental system for aging research (Fuchs, 2016). Skin wrinkling, hair greying, and hair loss are some of the most visible signs of aging, making them ideal subjects for investigation (Matsumura et al., 2016; Ge et al., 2020; Zhang et al., 2021). As an experimental system, hair follicle offers a particular advantage for experimental manipulation because functional decline of hair follicles is generally well-tolerated and does not cause detrimental damage to survival. Furthermore, hair follicle lineages are spatiotemporally well-defined (Blanpain and Fuchs, 2014), and these features facilitate the analysis of linking high-dimensional data, such as transcriptome and open chromatin data, to physiological changes of the tissue. Epigenetic and transcriptional regulation play important roles in governing cell fate and functions of hair follicles. For example, FOXC1 and NFATC1 transcription factors (TFs) have been implicated in the regulation of HF-SC aging, through the control of quiescence, cell adhesion and extracellular matrix (ECM) (Keyes et al., 2013; Lay et al., 2016; Zhang et al., 2021). Genetic deletion of DNA methyltransferase 1 (DNMT1), on the other hand, decreases HF-SC activation and leads to progressive alopecia (Li et al., 2012). Furthermore, aged HF-SCs showed increased niche stiffness (Wang et al., 2023), decreased open chromatin landscape and reduced ability to activate genes associated with self-renewal and differentiation (Koester et al., 2021).

With the advent of genomic tools, recent studies have begun to probe dynamic changes in the transcriptome and open chromatin dynamics in the hair follicles (Matsumura et al., 2016; Ge et al., 2020; Koester et al., 2021; Zhang et al., 2021). In particular, single-cell genomic tools have significantly advanced our understanding of cell state, cellular heterogeneity and aging-associated changes in hair follicle lineages. However, the atlas of hair follicle lineages during aging is incomplete. Previous studies have used single-cell RNA sequencing (scRNAseq) to profile skin from young (∼2 months old) and old (∼24 months old) mice, providing an initial comparison between the transcriptomes of young and old hair follicles (Ge et al., 2020; Zhang et al., 2021). However, aging, typically characterized by a gradual decline in physiological functions and regenerative capacity, does not necessarily proceed linearly. In hair follicles, the self-renewal of HF-SCs, which usually takes place during the anagen growth phase (Hsu et al., 2014), generates new HF-SCs. This process could effectively “reset” the aging process with each new round of cell division. It is important to note that the frequency of the anagen phase decreases with aging (Chen et al., 2014). Consequently, HF-SCs tend to remain in an extended quiescent state as aging progresses. Thus, to gain new insights into the hair follicle aging process, it is important to address several key questions: How does the hair cycle influence the aging trajectory? Do different cell types exhibit diverse trajectories and progressions during aging? What is the relationship between the transcriptome and the open chromatin landscape during aging?

In this study, we present a comprehensive single-cell analysis of the dynamics of the transcriptome and open chromatin landscape in distinct hair follicle lineages with a higher temporal resolution during aging. To facilitate the analysis of cell states at distinct hair cycle stages, we incorporated recently published scRNAseq datasets from postnatal day P38 (P38, catagen) (Zhang et al., 2021), and P53 (telogen) (Wang et al., 2023). Furthermore, we generated new scRNAseq datasets from mice aged 6 months (6 months), 12 months and 24 months. In addition, we also generated new scATACseq datasets from mice aged P28, 12 months and 24 months. Collectively, we have established a longitudinal single-cell atlas of both the transcriptome and open chromatin to probe physiological aging. The findings from this study provide an in-depth understanding of the cellular changes that occur in hair follicles during aging and contribute significantly to our overall understanding of the aging process.

## Results

### Longitudinal analysis of single-cell transcriptome during hair follicle aging

To capture the dynamic changes among the full spectrum of hair follicle cells during aging, we employed flow cytometry to sort epithelial cells, marked by H2B-GFP from *Krt14-H2B-GFP* mice, at postnatal day 38 (P38 male, synchronized catagen) (Zhang et al., 2021), P53 (female, synchronized telogen) (Wang et al., 2023), 6 months (female, mostly anagen HFs), 12 months (male, mixed telogen and anagen), and 24 months (female, mostly telogen). Single-cell RNA sequencing (scRNAseq) libraries were generated using the 10X Genomics Chromium platform (see Supplementary Table S1). The mixed use of male and female mice during aging was designed to reduce the possibility to capture sex-biased gene expression changes. After quality control and filtering, we obtained 14,091 single-cell transcriptomes, with an average of 2,797 genes (interquartile range 1,253) and 12,854 transcripts (interquartile range 9,402) detected in a single cell (Supplementary Figure S1A).

**FIGURE 1:**
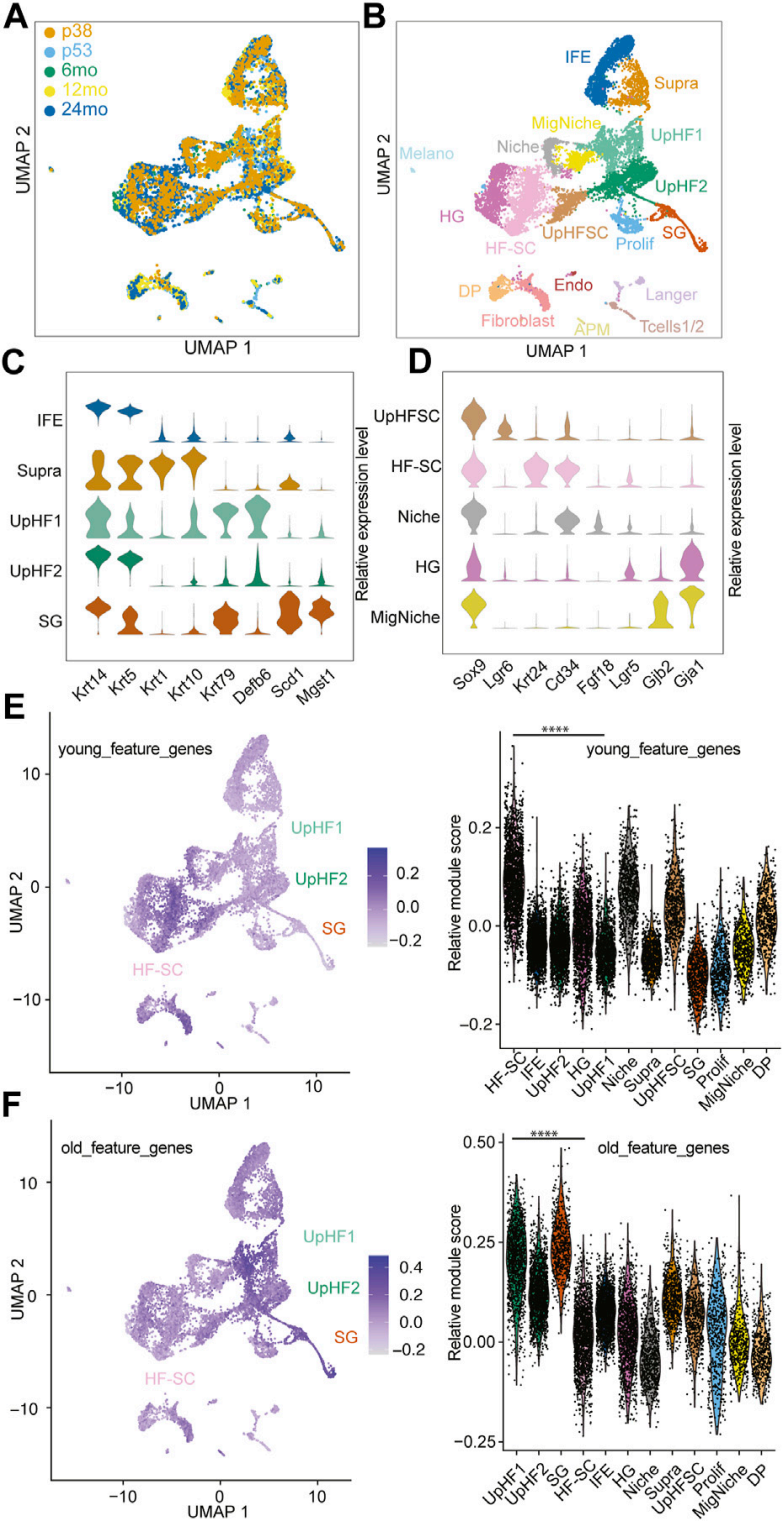
Single-Cell transcriptome of aging hair follicles. **(A)** Integration of scRNAseq samples from P38, P53, 6 months, 12 months, and 24 months. **(B)** UMAP visualization of all epidermal cell lineages. Color-coded by cell types. IFE, interfollicular epidermal basal cells; Supra, suprabasal cells; UpHF1/2, differentiated hair follicle cells in the upper portion; SG, sebaceous gland; UpHFSC, Lgr6+ HF-SCs; HF-SC, hair follicle stem cells; Niche, inner layer niche cells; HG, hair germ; MigNiche, migratory niche cells; DP, dermal papillae; Prolif, proliferating cells; Fibroblast, Fibroblast cells; Langer, Langerhans cells; Tcells1/2, T cells; APM, arrector pili muscle; Melano, melanocytes; Endo, endothelial cells. **(C,D)** Marker gene expression of different epithelial cell populations, interfollicular epidermal cells, upper hair follicles **(C)** and hair follicle stem cell compartment **(D)**. **(E,F)** Feature plot and violin plot of young **(E)** and old **(F)** feature genes sets. The young and old feature genes were genes enriched in young and old HF-SCs. The module scores were the average expression of aggregated feature genes on single cell level compared with control gene sets. The *p*-value of feature genes between UpHF1 and HF-SC population were calculated using wilcoxon rank sum test. ****: *p*-value <0.0001.

To integrate all the cells from different ages and reduce batch effects, we used unsupervised methods (Stuart et al., 2019) for integration and comparison (Figure 1A; Supplementary Figure S1A). We next applied unsupervised Louvain clustering based on the shared nearest neighbor graph (Hao et al., 2021) to batch-corrected samples (Figure 1B). Based on the differential expression analysis and known markers for well-characterized lineage markers for epithelial cells of the skin, we annotated the cell populations by using the top marker genes from each cluster (Figure 1B; Supplementary Figure S1B). Among the epidermal lineages, we successfully identified basal cells of the interfollicular epidermal lineages (IFE) (marked by Krt14 and Krt5), suprabasal cells of the IFE (supra, marked by Krt1 and Krt10), sebaceous gland lineages (SG, marked by Scd1and Mgst1), and hair follicle (HF) lineages in all samples (Figure 1C), as shown in the uniform manifold approximation and projection space (UMAP). However, because of our intention to enrich epithelial cells, the cell populations in the dermis, including fibroblast (marked by Dcn and Col1a1), arrector pili muscle (APM) (marked by Rgs5 and Acta2), dermal papillae (marked by Crabp1 and Lef1), immune cells (marked by Cd2 and Cd28), and endothelial cells (marked by Cdh5 and Pecam1) were captured in the datasets from P53, 6 months, 12 months, and 24 months samples but not the P38 sample (Supplementary Figures S1B, C). The P38 sample contained mostly epithelial cells with a minimal fraction of dermal cells and other non-epithelial cells because it was obtained by sorting Krt14-H2bGFP + epithelial cells (Supplementary Figure S1B). In epithelial cells derived from the bulge stem cell region (marked by high Sox9 expression), we successfully resolved the HF-SCs (marked by Krt24 and Cd34), inner bulge niche (Niche) (marked by Fgf18), upper HF-SCs (marked by Lgr6), and hair germ (HG) progenitors (marked by Lgr5) (Figure 1D). Interestingly, we also discovered a cell population related to the Krt6+ population (Hsu et al., 2011), which displayed a unique transcriptome with enriched gap junction gene expression, among others (Figure 1D). We named this population as migratory Niche (migNiche), which is distinct from the inner bulge niche. Gene ontology (GO) analysis indicated enriched programmed cell death, cell projection, supramolecular fiber organization, cell junction and actin cytoskeleton organization in the migNiche compared to inner bulge niche cells (Supplementary Figure S1D; Supplementary Table S2).

In previous aging studies of HFs, quantification of the SC population has typically relied on using cell surface markers specific to HF-SCs to quantify their numbers (Matsumura et al., 2016; Koester et al., 2021). However, several issues could interfere with the quantification. For example, it remains unclear whether these individual markers can accurately capture and quantify all HF-SCs during aging. In particular, aged HF-SCs may fail to express one or a few markers while still maintaining their SC functions. Therefore, a more unbiased analysis of cell population dynamics in HF lineages during aging is needed. In this study, we leveraged high-dimensional, scRNAseq datasets to annotate HF lineages, including HF-SCs, based on unsupervised clustering and curated annotation of a large set of genes. One challenge to quantify individual epithelial cell populations based on scRNAseq results was the variation in cell dissociation during sample preparation. For example, dermal cells were scraped before enzymatic digestion, resulting in less robust recovery of dermal cells in scRNAseq datasets (Supplementary Figure S1E). Because the 10X scRNAseq platform randomly samples up to 10,000 cells per assay, the percentage of cells from different populations could be affected by the different efficiency in cell dissociation and recovery. To minimize this effect, we restricted our compositional analysis to anatomically close lineages. We made the assumption that these closely situated epithelial cells are more likely to be exposed to the same dissociation conditions and thus maintain their relative proportions. Therefore, comparison of these local populations should more accurately reflect their composition during aging. In support of this view, our analysis revealed that the composition of IFE cells remained largely constant during aging (Supplementary Figure S1F). In contrast, the relative percentage of inner bulge niche cells in the bulge region decreased during aging (Supplementary Figure S1F). This observation was consistent with intravital live imaging data, which showed the reduced inner bulge niche in miniaturized HFs in aged mice (Zhang et al., 2021).

To investigate the patterns of gene expression changes in HF-SCs during aging, we extracted differentially expressed genes in HF-SCs from published bulk RNAseq data obtained from young (6 months) and old (24 months) mice (Ge et al., 2020) (Supplementary Table S3). When we mapped the genes enriched in young HF-SCs onto our integrated scRNAseq datasets, we found that HF-SCs had the highest expression of these genes associated with young HF-SCs (Figure 1E). Surprisingly, when we mapped the genes enriched in old HF-SCs onto the same integrated scRNAseq datasets, we found that Upper HF lineages, including epithelial cells from sebaceous gland and infundibular regions, showed the highest expression of these genes associated with old HF-SCs (Figure 1F). Further considering only the HF-SC populations, we validated that the old and young HF-SC gene signatures were indeed enriched in the corresponding conditions (Supplementary Figure S1G). These findings suggest a tantalizing possibility that aging HF-SCs acquire additional HF lineage features, in this case Upper HFs, while still maintaining their general HF-SC features. This observation is also consistent with spatial features of upward migration of HF-SCs during aging (Matsumura et al., 2016; Zhang et al., 2021). Collectively, these data provide molecular evidence that HF-SCs are gradually acquiring gene signatures associated with the upper hair follicle lineages during aging.

### Trajectory of hair follicle stem cell states during aging

**FIGURE 2:**
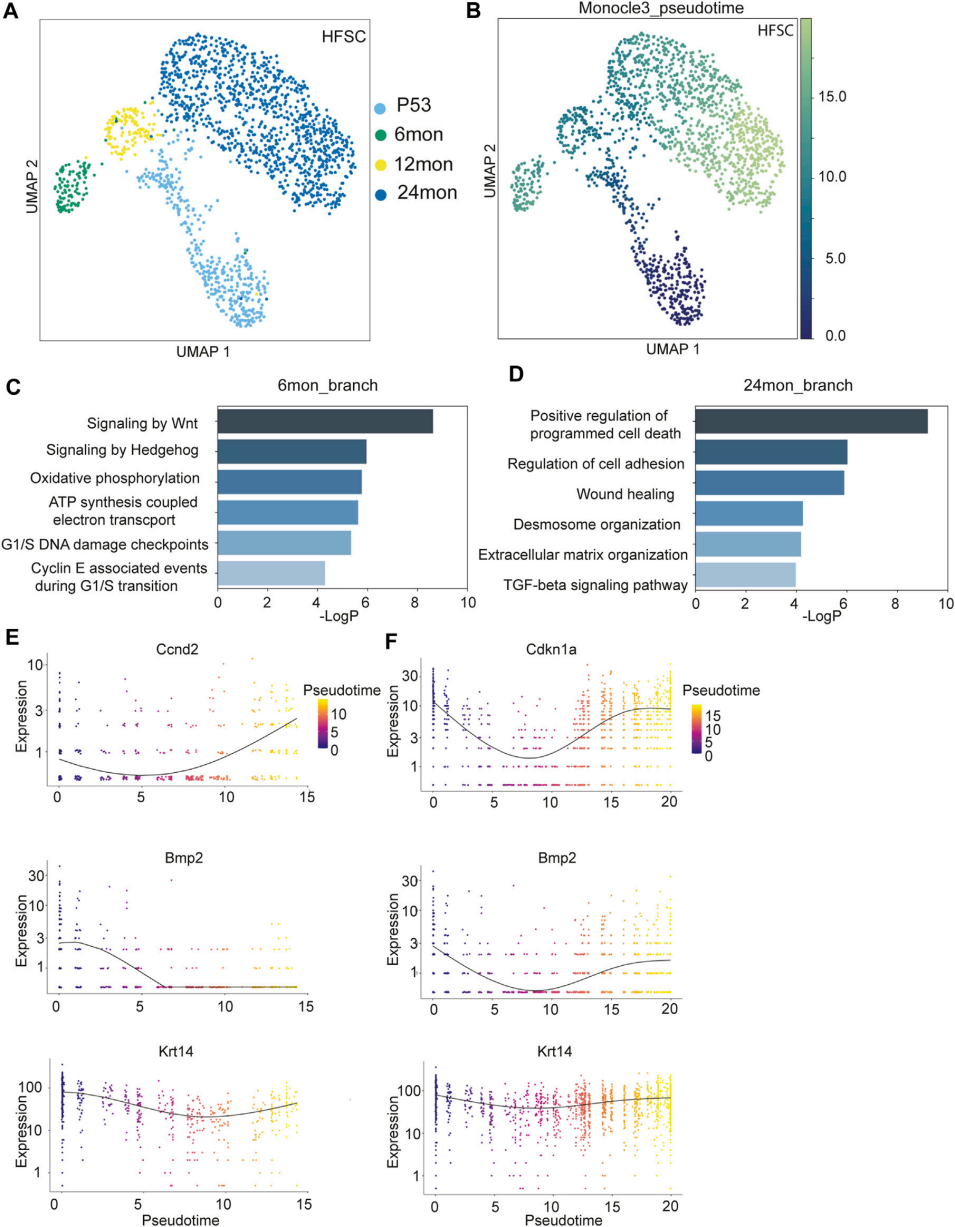
Transcriptomic analysis of HF-SCs aging. **(A)** UMAP visualization of HF-SCs, color coded by samples. **(B)** UMAP visualization of HF-SCs color-coded by pseudo-time. **(C,D)** Highly enriched gene ontology (GO) terms of 6 months **(C)** and 24 months **(D)** branch cells. **(E,F)** Selected gene expression plot along the pseudotime trajectory of 6 months **(E)** and 24 months **(F)** branch cells.

We next leverage our scRNAseq datasets across multiple time points to examine the trajectory of HF-SCs during aging. Because the P38 sample was derived from catagen, which is a transient stage between the growing anagen phase and the quiescent telogen phase, we only used this sample as a reference for catagen signatures. We extracted HF-SCs from the integrated scRNAseq datasets that were derived from P53, 6 months, 12 months, and 24 months samples. Upon re-clustering these HF-SCs, we observed a bifurcating trajectory with 6 months and 24 months cells residing at each branch (Figure 2A). This distinct pattern was notably different from those of upper HF lineages, which largely overlapped with each other (Supplementary Figure S2A). To minimize the possibility of algorithm-dependent artifacts, we used an independent, partition-based graph abstraction (PAGA) algorithm (Wolf et al., 2019) that preserves global topology of scRNAseq dataset to generate the cell trajectory. We still observed a similar pattern of bifurcation between 6 months and 24 months datasets when we obtained the single cell embedding results (Supplementary Figure S2B). In addition, since these clusters were derived from the same sample set, it is likely that the observed differences were caused by cluster-specific aging trajectories rather than systemic differences, such as sex bias. Given that both 6 months and 24 months samples were female, this further argued against the idea that the bifurcating pattern could be due to sex biases within the samples.

To better understand the transcriptomic dynamics along these two trajectories, we used Monocle3 (Cao et al., 2019) to order the HF-SCs in pseudo-time with P53 cells as the root (Figure 2B). We separated the cells into two groups based on their branches in the trajectory (Supplementary Figures S2C, D) and compared the transcriptome of 6 months and 24 months samples on each branch. GO analysis for genes associated with the 6 months sample uncovered enriched signaling pathways, including Wnt/hedgehog signaling, which are associated with anagen HF-SC activation and proliferation (Hsu and Fuchs, 2012; Hsu et al., 2014), metabolic pathways, including oxidative phosphorylation and ATP synthesis coupled electron transport, and cell cycle G1/S checkpoints (Figure 2C; Supplementary Table S4). These signatures were consistent with the notion that the 6 months sample was isolated from mostly anagen skin. On the other hand, the GO signatures of the 24 months HF-SCs were enriched for positive regulation of programmed cell death, altered extracellular matrix and cell adhesion and wound healing (Figure 2D; Supplementary Table S4). Furthermore, these aged HF-SCs were enriched for pro-inflammatory signals such as TNFα and IL2-Stat5 signaling (Supplementary Figure S2E, F). These data reveal an elevated state of inflammation in aged skin. It is plausible that this activated immune program could be responsible for clearing out escaped HF cells in aged skin (Zhang et al., 2021). Additionally, these data also indicate that the stages of the hair cycle, particularly the anagen phase during which HF-SCs self-renew, could influence the cell state and transcriptome of HF-SCs.

To probe more deeply into the transcriptomic changes along the bifurcating trajectory, we examined the 6 months branch, which represent the telogen (P53) to anagen (6 months) transition, and the 24 months branch, which represent the aging trajectory of typical telogen HF-SCs, by using Monocle3 (Cao et al., 2019) (Supplementary Figures S2C, D). Along the 6 months trajectory, cyclin genes responsible for the G1/S transition (Ccnd2) was upregulated, while cell cycle inhibitor (Cdkn1a) showed reduced expression, further corroborating the status of activated HF-SCs in the 6 months sample (Figure 2E). In addition, Wnt signaling components (β-catenin, Fzd2, Tcf7l2) gradually increased expression (Supplementary Figure S2G) while Bmp2 levels were reduced as expected for anagen HF-SCs (Plikus et al., 2008) (Figure 2E). In both trajectories, Krt14 expression level was relatively stable and used as internal control. To gain insights into global gene expression patterns, we employed Monocle3 to categorize the genes into distinct clusters using Louvain community analysis. This analysis identified two clusters demonstrating different patterns along the temporal trajectory (Supplementary Figures S3A, B) on the 6 months branch (Supplementary Table S5). Cluster1, containing 2,614 genes, showed a consistent increase in expression levels along the trajectory, whereas Cluster2, with 1,520 genes, exhibited a diminishing expression pattern. From these clusters, we discerned that gene signatures associated with metabolic stress (Cluster 1, Supplementary Figure S3C) and decreased cell adhesion and tight junction (Cluster 2, Supplementary Figure S3D) emerge as early as the 6 months mark in HF-SCs.

In the 24 months aging trajectory, expression of genes associated with quiescence, such as Bmp2 and Cdkn1a, was elevated toward the end point of 24 months (Figure 2F), consistent with the notion that aged HF-SCs mostly rest in the prolonged quiescent state (Keyes et al., 2013; Ge et al., 2020; Zhang et al., 2021). The co-expression analysis revealed two distinct and oscillating patterns along the trajectory from P53 to 12 months–24 months. Cluster1, containing 2,515 genes, displayed a gradual increase in expression followed by a decrease (Supplementary Figure S3E), whereas Cluster2, with 1,743 genes, showed a pattern with an initially high expression level followed by downregulation and subsequent recovery (Supplementary Figure S3F; Supplementary Table S6). Interestingly, the cluster1 genes were enriched for GO terms similar to those of the 6 months branch, including oxidative phosphorylation, Wnt signaling and cell cycle checkpoints (Supplementary Figure S3G). Furthermore, Runx1 transcriptional regulation was increased in Cluster1 genes, which has been previously reported to promote activation of HF-SCs (Hoi et al., 2010; Lee et al., 2013). This analysis also revealed a strong enrichment for reactive oxygen species (ROS) in Cluster1 genes, which was not evident in direct comparisons (Supplementary Figure S3G). Given the similarity between the genes of Cluster1 and those of the 6 months (anagen) branch, this analysis suggests that anagen-associated gene signatures gradually diminish during the prolonged telogen phase associated with aging. The cluster2 genes, which demonstrated a pattern of reduced expression followed by recovery, were enriched for the regulation of cell adhesion and migration (Supplementary Figure S3H). Prior comparisons between P53 and 24 months samples have also revealed a downregulation of cell adhesion and the extracellular matrix (ECM) (Ge et al., 2020; Zhang et al., 2021). These dynamic patterns, as detected by our analysis with higher temporal resolution, likely indicate an active yet insufficient effort by HF-SCs to restore cell adhesion and the ECM microenvironment during aging. Taken together, these results underscore the existence of a dynamic, rather than linear, pattern in the gene expression program of HF-SCs during aging.

### Trajectory of inner bulge niche cell states during aging

**FIGURE 3:**
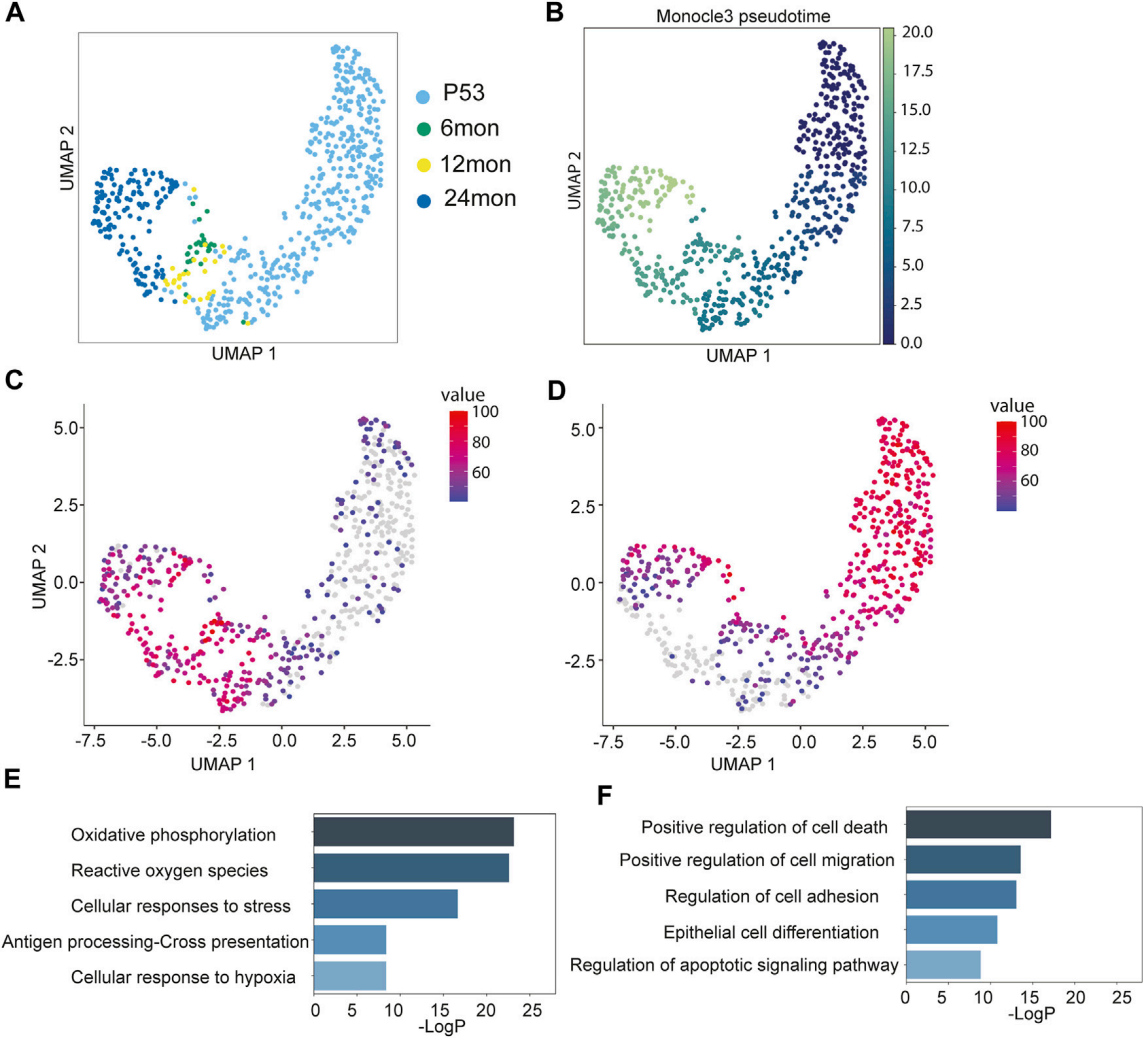
Aging trajectory of the Niche cells. **(A)** UMAP visualization of Niche cells during aging, color-coded by samples. **(B)** Monocle3 pseudo-time plots of aging Niche cells color-coded by pseudo-time values. **(C,D)** Aggregated expression of genes modules in different co-expression modules along the aging Niche cells. **(E,F)** Enriched GO terms of gene modules corresponding to **(C)** and **(D)**.

The analysis of inner bulge niche cell composition revealed a reduction in their population during aging (Supplementary Figure S1E). To investigate the cellular state dynamics of the Niche cells, we subsected the Niche population from all samples and re-clustered them. Interestingly, unlike the bifurcating patterns observed in HF-SCs, the Niche cells exhibited a linear progression from P53 cells to 24 months cells (Figure 3A). In support to these results, the force-directed graph also displayed a similar linear progression (Supplementary Figure S4A). This linear trajectory, in contrast to the bifurcating pattern observed in HF-SCs, suggests that the aging trajectory of the Niche cells is different from those of HF-SCs.

To further determine transcriptomic dynamics of Niche cells, we employed Monocle3 to generate the pseudo-time trajectory and calculate the pseudo-time value for individual Niche cells. Consistent with the linear progression pattern, the 24 months cells had the highest pseudo-time values, followed by the 6 months/12 months cells that had intermediate values, whereas P53 cells had the lowest pseudo-time value as the root (Figure 3B). Because the pseudo-time analysis recapitulated the physiological age of the Niche cells, this enabled us to infer the changes along the aging process. The analysis of covaried gene expression changes identified three distinct patterns. Among them, Cluster1, containing 1,699 genes, and Cluster2, containing 1,581 genes, turned on or off later in the trajectory, respectively (Figures 3C, D). In contrast, Cluster3, containing 158 genes, largely maintained gene expression across all stages (Supplementary Figure S4B). The Cluster1 genes, which were elevated during aging, showed an increase in oxidative phosphorylation, reactive oxygen species (ROS), stress response, antigen presentation and hypoxia (Figure 3E). This trajectory suggests that the Niche cells may accumulate gene expression associated with stress response. The Cluster2 genes, which diminished during aging, were enriched for genes associated with cell death, cell migration, cell adhesion and epidermal differentiation pathways (Figure 3F).

Unlike HF-SCs, whose gene expression patterns seem to fluctuate during aging, the Niche cells appear to accumulate gene expression associated with cellular stress. These signatures could indicate a relatively fragile state of the Niche cells that continuously declines during aging. These observations position the Niche cells as promising targets for future functional investigation into HF aging.

### Integrative analysis of single-cell RNA sequencing and single-cell ATAC sequencing

**FIGURE 4:**
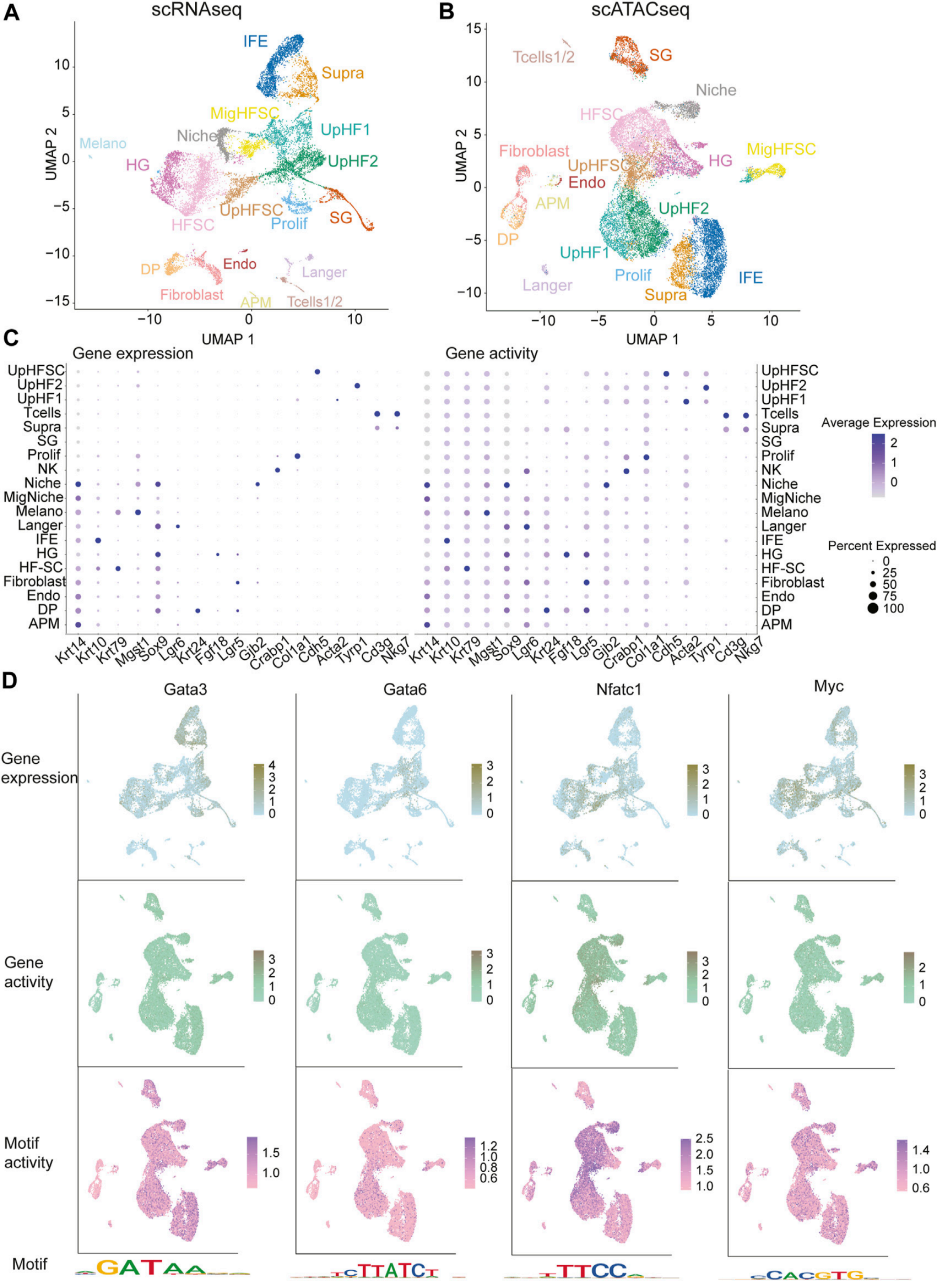
Integration of scRNAseq and scATACseq data. **(A,B)** UMAP visualization of integrated scRNAseq and scATACseq data, color-coded by cell populations. **(C)** Dotplot of marker genes expression in scRNAseq (left) and inferred gene activities in scATACseq (right). Gene expression levels were indicated by color intensity. Dot size represents the percentage of cells with the inferred activities. **(D)** Gene expression, inferred gene activities, motif activities and motif plots of lineage specific transcription factors.

scRNAseq has a limitation of poor detection rate for lowly expression genes, including transcription factors (TFs), which are critical for cell fate specification and cell state maintenance. In contrast, single-cell ATAC sequencing (scATACseq) can infer transcription factor activity based on patterns of chromatin accessibility (Buenrostro et al., 2013; Wang et al., 2016; Fan et al., 2018; Zhang et al., 2021). To understand the multi-modality of HF aging, we performed scATACseq on P28 (young, male), 12 months (middle age, male), and 24 months (old, female) samples. After initial processing (Supplementary Figures S5A, B; Supplementary Table S1), we selected cells with a number of fragments overlapping peaks between 3,000 and 100,000, with more than 40% reads in peaks, and a blacklist ratio less than 0.025. Furthermore, we calculated the transcriptional start sites (TSS) enrichment signal and nucleosome signal per cell by using signac (Stuart et al., 2021) to filter out cells with low TSS and high nucleosome signal. We sequenced approximately 20,492 cells from all samples (Figures 4A, B) with a similar sequencing saturation rate (63.6%–64.1%). Interestingly, P28 samples had approximately 20 K fragments in peak regions per cell after filtering, while 12 months and 24 months samples each had around 10 K fragments with the similar sequencing depth. This finding unveils the widespread closure of chromatin accessible region as early as 12 months in epithelial cells of the skin. This corroborates similar observations detected by bulk ATAC-seq in a recent study (Koester et al., 2021).

To capture high-confidence peaks in all samples for analysis, we pooled all cells from each sample and assembled them into a collective bulk ATAC dataset for peak calling. In total, we detected 178,454 peaks in P28, 111,246 peaks in 12 months, and 145,689 peaks in 24 months samples, confirming the reduction of open chromatin regions in middle-aged and old samples. To facilitate the integration of all scATACseq data, we combined peaks across all samples. To find integration anchors from all samples, we applied signac (Stuart et al., 2021) to project all samples into a shared low-dimensional space using reciprocal latent semantic indexing (LSI). In this processing, we excluded the first component because it was highly correlated with sequencing depth (Supplementary Figures S5C, D).

As scRNAseq datasets have been successfully employed to annotate all epithelial cell populations of adult skin (Joost et al., 2016; 2020), we used scRNAseq as a reference and mapped the scATACseq data onto it to aid cell type identification (Figures 4A, B; Supplementary Figures S5E–G). The comprehensive collection of samples obtained from comparable stages allowed us to perform an integrative analysis of gene expression using scRNAseq data, gene activities by counting open chromatin fragments from scATACseq data overlapping with the gene body and located in the upstream region of the TSS, and motif enrichment by conducting TF motif analysis in scATACseq datasets. Remarkably, a strong Pearson correlation was observed between marker gene expression obtained from scRNAseq and predicted gene activity derived from scATACseq for each cluster (Supplementary Figure S5H), validating our integrative analysis. Interestingly, while open chromatin signatures (dot size) showed similar signals across the epithelial cells, predicted gene activities (color) showed a stronger correlation with unique gene expression levels in each cell population (Figure 4C, right panel). These data suggest that open chromatin signatures *per se* do not predict transcriptional output, but predicted gene activities serve as a more reliable indicator of transcription. We note that proliferating cells in scRNAseq data failed to map onto the scATACseq space, a finding that has been reported previously (Trevino et al., 2021). This result further illuminates the disparity between open chromatin signatures, transcriptional activity, and gene expression levels.

Next, we calculated lineage-specific TF motif activity using Chromvar (Schep et al., 2017). As expected, gene expression levels, predicted gene activity, and motif activity all demonstrated enrichment for lineage-specific TFs. For IFE and UpHF populations, Gata3, Gata6, Jun, and Grhl1 all exhibited high expression levels along with high motif activities (Figure 4D; Supplementary Figures S6A, B), which is consistent with their biological functions in these skin lineages. For the bulge HF-SCs, Sox9, Nfatc1, and Lhx2 signals were significantly enriched (Figure 4D; Supplementary Figure S6C). We also noted that motif activities and gene expression levels showed higher lineage specificity than the predicted gene activities, underscoring the importance of functional measurement of gene expression. Interestingly, even TFs that were lowly expressed and not robustly detected in scRNAseq, such as Rbpj, displayed strong signals in motif activities, as shown in Supplementary Figure S6D.

### The dynamics of chromatin accessibility during hair follicle aging

**FIGURE 5:**
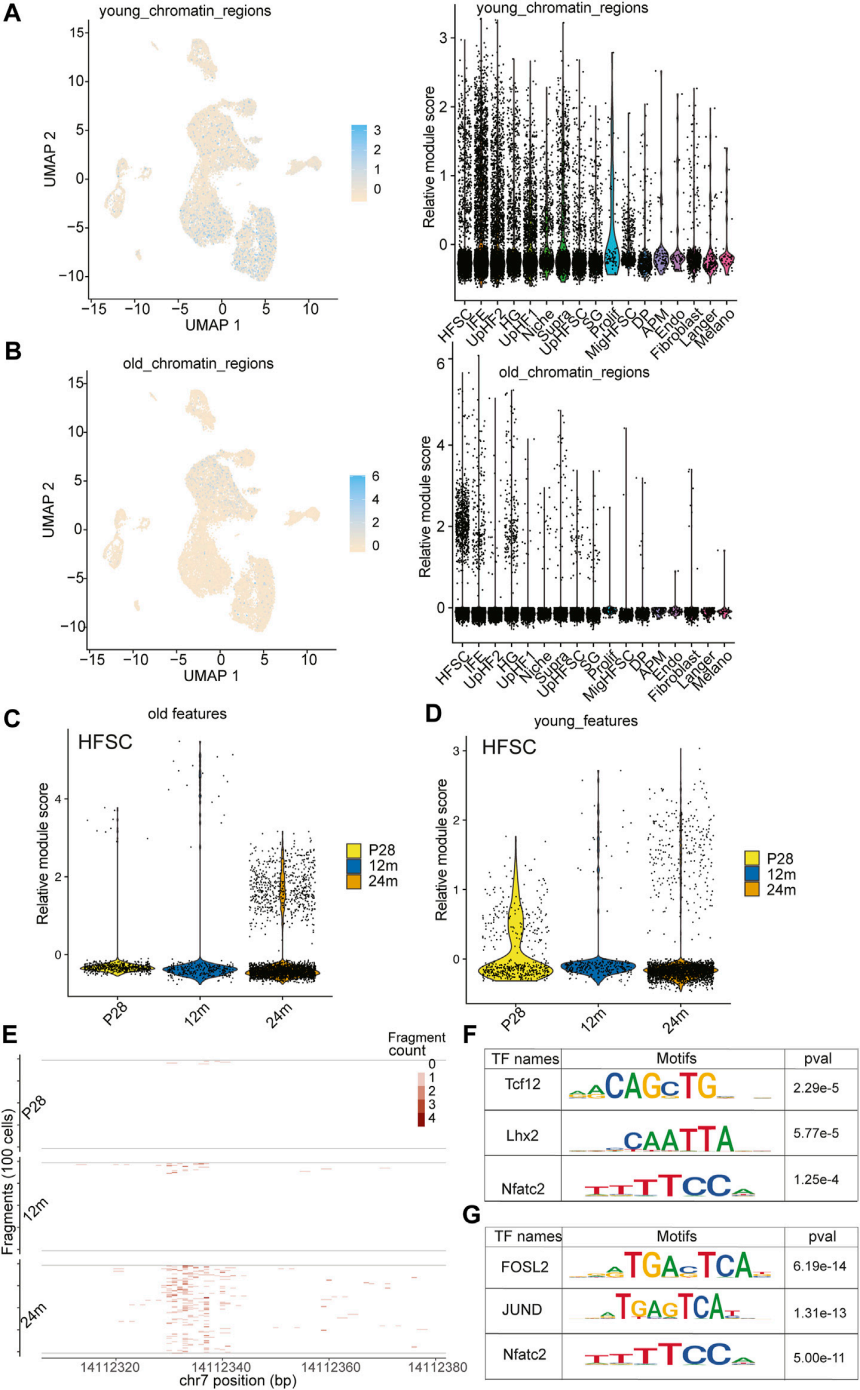
Open chromatin dynamics during HF-SC aging. **(A)** Feature plot and violin plot of open chromatin regions enriched in young HF-SCs. **(B)** Feature plot and violin plot of open chromatin regions enriched in old HF-SCs. **(C,D)** Violin plot of young and old open chromatin features in HF-SCs of different samples. **(E)** Example tileplot of chromatin regions gradually open during aging. **(F,G)** Enriched motifs in young and old HF-SC open chromatin regions.

To investigate the epigenetic changes that occur during HF aging, we analyzed published young and old bulk ATACseq datasets to identify differentially accessible regions (Koester et al., 2021) (Supplementary Figure S7). This analysis led to an unexpected discovery when we mapped these differentially accessible regions onto our scATACseq data. The open chromatin regions that were more accessible in young HF-SCs were enriched across all epithelial populations, including both stem cells (SCs) and differentiated cells, as shown in Figure 5A; Supplementary Figure S6E. However, the open chromatin regions that were more accessible in old HF-SCs were primarily open in HF-SCs, particularly in HF-SCs from old mice, as shown in Figure 5B; Supplementary Figure S6F. This finding suggests that HF-SCs gradually close some chromatin regions that are universally open in epithelial lineages during aging (Figure 5C), possibly indicating the limited differentiation potential in old HF-SCs. Conversely, the chromatin regions that became open in old HF-SCs were specific to these cells, likely reflecting their unique epigenetic changes during aging (Figure 5D).

To better understand the transcriptional regulation underlying these chromatin changes, we investigated the motif enrichment of the differentially accessible regions (Figure 5E). Interestingly, the regions in old HF-SCs were enriched for the motif of Lhx2 (Figure 5F), which is required for quiescence control and cell fate maintenance of HF-SCs (Folgueras et al., 2013), whereas the regions enriched in young HF-SCs harbor Jun and Fosl2 motifs (Figure 5G). Overall, these findings shed light on the chromatin accessibility dynamics during HF aging and provide a genomic reference to study the maintenance of HF-SC functions during aging.

## Discussion

### Cell type specific changes of transcriptome during hair follicle aging

In this study, we have profiled single-cell transcriptome of the skin from young, middle-age and old mice. This strategy has allowed us to gain new insights into transcriptome dynamics of HF-SCs through the hair cycle as well as during aging. Previous studies have examined the aging process of HF-SCs by profiling FACS purified HF-SCs with bulk RNAseq (Matsumura et al., 2016) or the total skin populations by using scRNAseq of young and old skin (Ge et al., 2020; Zhang et al., 2021). By introducing intermediate samples to reflect the hair cycle differences and the progression of aging, our results have revealed unexpected results that different cell populations, such as HF-SCs and the inner bulge niche cells, have different changes in their transcriptome during aging. The aging-associated alterations in the transcriptome of HF-SCs and their inner bulge niche cells are found to be more pronounced than in the Upper HF lineages. This observation could be attributed to the more dynamic changes experienced by HF cells during the hair cycle, compared to the relative stability of the Upper HF cells. HF-SCs, located in the bulge area of the hair follicles, are known for their ability to undergo cyclic phases of quiescence and activation in response to various signals but also experience prolonged quiescence during aging (Yi, 2017). As such, the transcriptomic profile of these cells is expected to show considerable variations over time, in particularly the association of the hair cycle. Indeed, we detected bifurcating trajectories of HF-SC cell state, likely caused by the hair cycle stages in addition to the *bona fide* aging process (Figures 2A, B). Interestingly, between HF-SCs and the niche cells, the niche cells showed a more linear progression of transcriptomic changes whereas HF-SCs show more dynamics in their transcriptome, some of which may be explained by the hair cycle stages. These findings suggest that different cell lineages respond to the aging process differently. The concept of differential aging process, the underlying mechanism and their implication in aging should be examined carefully in functional studies in the future.

### Rethinking hair follicle aging

The paradigm of SC senescence and exhaustion has been central to our understanding of aging (López-Otín et al., 2023). However, HF-SCs maintain their proliferative capacity and even showing signs of rejuvenation when exposed to a youthful dermis during aging (Ge et al., 2020). These intriguing observations suggest that the microenvironment plays an important role in determining the state and function of HF-SCs. Indeed, our recent study revealed that cell adhesion and ECM deposition by HF-SCs, controlled by FOXC1 and NFATC1 TFs, are critical for the integrity of the bulge HF-SC compartment (Zhang et al., 2021). Consistent with this view, our comprehensive longitudinal analysis uncovers that HF-SCs largely maintain their identity over time, while the inner bulge niche cells, which are known to regulate HF-SC function (Kimura-Ueki et al., 2012; Lay et al., 2016), undergo more profound and irreversible changes in their transcriptome. These changes in the inner bulge niche cells could potentially disrupt the microenvironment necessary for the maintenance and function of HF-SCs, signifying a form of “indirect aging” where the SCs performance is compromised not by their intrinsic aging but by the aging of their niche. Furthermore, our scRNAseq analysis has revealed an intriguing trend: aging HF-SCs begin to adopt transcriptomic signatures reminiscent of the upper HF lineages. This finding provides new insights into the shift in the identity of aging HF-SCs, potentially representing a form of “lineage infidelity” or “lineage drift” that may affect their functional integrity.

Complementing these transcriptomic changes, our scATACseq analysis has revealed alterations in the open chromatin landscape of aging HF-SCs. We observe reduced open chromatin regions in aging HF-SCs, which may reflect a compromise in their differentiation potential. Concurrently, we see an increase in open chromatin regions, which are likely associated with prolonged quiescence. These new insights thus provide a molecular basis to further examine and potentially reverse the aging process in experimental studies.

### Limitation of the study

One of the persistent challenges in longitudinal, single-cell studies is the batch effect. These variations can lead to spurious results, making it challenging to distinguish between true biological differences and technical artifacts. Although we have made considerable efforts to minimize these effects in our study, the limited number of samples for each time point may have inevitably introduced some batch-related variability. Another challenge is the potential confounding effects related to sex. Male and female mice may exhibit distinct cellular and physiological processes during aging. In our current study, we used a mixed cohort of male and female mice. While this approach has the advantage of capturing a broader spectrum of biological phenomena, it also introduces additional complexity. Differences observed might be due to sex-specific effects rather than, or in addition to, aging *per se*. Our study, therefore, underlines the importance of addressing these issues in future research.

## Methods

### Mice

All experiments were carried out following IACUC-approved protocols and guidelines at CU Boulder and Northwestern University. Mice were housed according to guidelines of the IACUC at a pathogen-free facility at University of Colorado at Boulder and at Northwestern University Feinberg School of Medicine. The K14-H2BGFP (E.Fuchs, Rockefeller University) mouse line was used for sorting epidermal cells. The samples used for scRNA sequencing include both male and females, P38, male; P53, female; 6 months, female; 12 months, male; 24 months, female. For scATAC samples, P28 and 12 months samples were male and 24 months sample was female.

### Tissue processing and fluorescence-activate cell sorting

Mice were euthanized and collected for dissection. We first shaved the hair coat and applied Nair hair removal lotion (Amazon, 22,339) for around 3 min. After wiping off the lotion and washing away leftover hair shafts, back skin was dissected, and subcutaneous fat was removed using a blade. As small part of the skin sample was embedded in OCT, and the remaining skin sample was minced and incubated with 0.25% collagenase (Worthington, LS004188) in 4–6 mL 1X HBSS buffer at 37°C for 2 h with rotation. A 5-mL serological pipet was used to further separate the epidermis from the dermis at the 1 h incubation time. After collagenase treatment, we added 10 mL cold PBS and centrifuged the sample at 400 g for 10 min at 4°C. The pellet was resuspended with pre-warmed 0.25% trypsin-EDTA (Gibco) for 8 min at 37°C, and the digestion was immediately blocked by adding 10 mL cold 1XPBS with 3% chelated PBS. Cells were incubated with appropriate antibodies for 1 h on ice. DAPI was used to exclude dead cells. Cells from K14-Cre-based experiments were isolated by enriching for DAPI-K14-H2BGFP + epidermal cells and DAPI-K14-H2BGFP- dermal cells.

### Bulk RNAseq analysis

Bulk RNAseq data from Fuchs group (Ge et al., 2020) (accession number GSE124901) were downloaded using SRA-toolkit (version 2.8.0) fastq-dump. The fastq files were then mapped to mouse genome (mm10) using Hisat2 (version 2.1.0) with options -p 32 --rna-strandness RF to generate sam files. The samtools (version 1.3.1) were used to convert samfile to bamfile. The final counts files were generated by htseq (version 0.9.1) with options -t exon -i gene_id --stranded = reverse -f.

### scRNAseq library preparation

Single cells from different age groups were collected from a flow cytometry-sorting machine with cell surface proteins and H2BGFP signals such that epidermal cells and hair follicle cells were at a 1:3 ratio. For each sample, around 2,000–5,000 cells were used for scRNAseq libraries. Libraries were prepared using the 10X Chromium Single Cell 3ʹ GEM, Library & Gel Bead Kit version 3 chemistry (PN-1000110). In brief, FACS-sorted cells were diluted to suggested concentration. The single cell suspension, single cell 3′gel beads and the reverse transcription mix were then incubated to generate gel beads emulsion and barcode. The resulting cDNA were pooled and amplified followed by library construction. The libraries were quality checked on an Agilent Bioanalyser 2,100 using the high sensitivity DNA kit (5067–4626).

### Upstream analysis of scRNAseq data

The Cell Ranger Single-Cell Software Suite was used to perform barcode processing and single-cell gene counting on demultiplexed raw sequencing data. The scRNAseq reads were mapped to the mouse (mm10) reference genome and quantified using cellranger count (version 3.0.1). The resulting barcodes, features and matrix files were used for downstream analysis. The scRNAseq output files from Fuchs group (Ge et al., 2020) was downloaded directly through GEO (accession number GSE124901).

### Downstream analysis of scRNAseq data

The barcodes, features and matrix files were loaded into Seurat (version 4.0.5) R (version 4.1.0) package for further analysis. The following criteria were used to filter out low quality cells:

200< nFeature_RNA<5,000 and percentage of mitochondrial <15%. After filtering, we have 2,109 cells for P38 sample, 3,827 cells for P53, 1847 cells for 6 months, 3,236 cells for 12 months and 3,072 cells for 24 months.

The count data was log-normalized and scaled to 10,000. The PCA analysis was based on top 2,000 variable genes. The nearest neighbors were computed based on the Euclidean distance in PCA space. To cluster the cells, the Louvain algorithm was implemented. Uniform manifold approximation and projection n (UMAP) was used for non-linear dimension reduction. To integrate all scRNAseq samples, the FindIntegrationAnchors function from Seurat takes all the Seurat object and identify anchor by utilize canonical correlation analysis (CCA) as initial dimension reduction. The integrated datasets were then scaled and clustered. To annotate each cluster, the FindAllMarkers function were used to identify cluster specific marker genes. The integrated datasets were then subsected based on cell types.

For scanpy (Wolf et al., 2018) (version 1.7.0) analysis, the Seurat object were converted to h5ad file using R packages (SeuratDisk, SeuratData). For PAGA analysis, sc.tl.paga was first used to compute the connectivity of clusters followed by sc.pl.draw_graph to get single-cell embeddings that are faithful to global topology.

For module score analysis, the differential expression gene lists from young and old HF-SC bulk RNAseq were imported in R. The young and old features include gene with basemean value greater than 600 and padj value less than 0.05. The module score was then computed using AddModuleScore function. The Wilcoxon rank sum test were used to compute significance between cell populations and samples.

For trajectory analysis, the pre-clustered cells from Seurat object were the converted to monocle object maintaining the UMAP information. The single-cell trajectory was then constructed by setting P53 samples as root and pseudo-time value was calculated by ordering cells along the trajectory. The specific branch of interest was chosen by setting the root cells as P53 and ending nodes as cells on the end of the branch. To find co-regulated genes modules along specific trajectory, the find_module_df function was used with resolution as 0.0001. Aggregated gene expression from different modules were then plotted. Note, the individual genes find in different modules might not reflect the aggregated pattern.

### scATACseq library preparation

The single cells solution of skin cells was generated the same as scRNAseq. In total, 10,000 cells from each sample were used for scATAC-seq preparation. Libraries were prepared using the 10X Chromium Single Cell ATAC Library & Gel Bead kit (PN-1000110).

In brief, cell nuclei were isolated, and nuclear suspension were incubated in a transposition mix to fragment DNA and add adaptor sequence to the end of DNA fragments. Single-nucleus resolution was achieved using 10X barcoded gel beads, partitioning oil and a master mix on a Chromium Chip E. Libraries were constructed using a 10X sample index plate and double size selected from 150 bp to 1000 bp. The final libraries were quality-checked with bio-analyzer before sequencing.

### Upstream analysis of scATACseq data

FASTQ files were collected from the sequencing facility and concatenated together. We used cellranger-atac (version 3.0.1) counts with the reference genome downloaded from the 10X Genomics website. The P28 sample was re-sequenced from the same library previously published and concatenated for this study. The 24 months sample was re-analyzed using cellranger-atac reanalyze to filter out low quality cells.

### Downstream analysis of scATACseq data

The fragment file, peak file and single-cell metadata were loaded into signac (Stuart et al., 2021) for downstream analysis. The peak file generated from cell-ranger was first used for quality control. The following criteria were used to filter out low quality cells:

3,000 < peak region fragments <100,000
percentage of reads in peaks >40%
blasklist ratio <0.025
nucleosome signal <4
TSS enrichment >2

After filtering, we have 6,784 cells for P28 sample, 5,263 cells for 12 months sample and 8,445 cells for 24 months sample.

The gene activity matrix was then calculated and added to the seurat object.

For integration, we first generate combined peaks containing peaks from all samples. To do this, the R1 and R3 reads file from the single cell sequencing were treated as bulk samples and then mapped to mouse reference genome and called peaks. The peaks files were then merged using bedtools (Quinlan and Hall, 2010). The combined peaks file was then used on all samples to regenerate the matrix counts file overlapping the genomic regions. The same filtering criteria were used followed by normalization, dimension reduction and clustering. For the UMAP dimension reduction, we excluded dimension 1 suggested by signac (Stuart et al., 2021). The samples were then first merged followed by standard processing. The integration anchors were then found followed by integration.

For the integration of scRNAseq and scATACseq, the processed scRNAseq and scATACseq data were loaded in Seurat. The gene activity matrix calculated from scATACseq and the variable genes from scRNAseq were used to find anchors. The cell annotation label from the scRNAseq were then transferred to scATACseq. The scRNAseq and scATACseq were then co-embedded for visualization. For Pearson correlation analysis, the predicted gene activity from scATACseq and gene expression from scRNAseq were averaged by each cell population. The top 200 variable genes from scRNAseq data were used to calculate the Pearson correlation score.

For the differential peaks, the annotated scATACseq data were used to find all markers based on cell types. For motif analysis, the JASPAR 2020 (Fornes et al., 2020) and TFBSTools (Tan and Lenhard, 2016) were loaded. The motif activities were calculated by chromvar (Schep et al., 2017). The differential activities were then computed by FindAllMarkers function.

### ATAC-seq and motif analysis

ATAC-seq reads (paired end) were aligned to the mouse genome (NCBI37/mm10) using Bowtie 2 (version 2.2.3) (Langmead and Salzberg, 2012). Duplicate reads were removed with Picard tools (http://broadinstitute.github.io/picard/).

Mitochondrial reads were removed, and peaks were called on each individual sample by MACS (version 2.0.9) (Zhang et al., 2008). Peaks from different ATAC-seq samples were merged for downstream analysis. *De novo* motif discovery was performed using HOMER (Heinz et al., 2010). Motif scanning was performed with MEME (5.0.3) (Bailey et al., 2015). BED files were converted to FASTA files by bedtools getfasta (Quinlan and Hall, 2010), and motifs discovered by HOMER were used to scan for instances in open-chromatin regions. HOMER motifs were also converted to MEME format with the R package from GitHub\\ (href{https://gist.github.com/rtraborn/e395776b965398c54c4d}). For IGV visualization (Robinson et al., 2011), we first concatenated all peaks from samples of interest and converted them into a GTF file, counted the number of reads mapped in peaks and then normalized all samples using “bedtools genomecov -scale” to obtain bedGraph files. Igvtools toTDF was used to obtain TDF files for final visualization.

### Differential expression analysis

The counts files were calculated from the BAM alignment file by HTSeq (Anders et al., 2015). Differentially expressed genes were determined using DEseq2 (Love et al., 2014) with an adjusted *p*-value cutoff of 0.05. GO analysis was performed using Metascape (Zhou et al., 2019). Selected GO terms were from Metascape results along with the gene list.

For gene set enrichment analysis (Subramanian et al., 2005), the differential expressed genes were ranked by expression value and fold change.

## Data Availability

The datasets presented in this study can be found in online repositories. The names of the repository/repositories and accession number(s) can be found in the article/Supplementary Material. Datasets can also be found in the NCBI GEO database (https://www.ncbi.nlm.nih.gov/geo/) under accession number GSE227784.
