# Supplementary material for: Single cell analysis of transcriptome and open chromatin reveals the dynamics of hair follicle stem cell aging
Source: Front Aging. 2023 Jul 3;4:1192149. doi: 10.3389/fragi.2023.1192149 (PMC10350644; doi:10.3389/fragi.2023.1192149)
Supplement: Supplementary file 4 [file DataSheet1.pdf]

Supplementary Material for

Single cell analysis of transcriptome and open chromatin reveals the dynamics of hair follicle stem cell aging

Chi Zhang[1,5], Dongmei Wang[1,2,3], Robin Dowell[4] & Rui Yi[1,2,3]*

[1]Department of Pathology, Northwestern University Feinberg School of Medicine; Chicago, IL USA 60611

[2]Department of Dermatology, Northwestern University Feinberg School of Medicine; Chicago, IL USA 60611

[3]Robert H. Lurie Comprehensive Cancer Center, Northwestern University Feinberg School of Medicine; Chicago, IL USA 60611

[4]BioFrontiers Institute, University of Colorado Boulder, Boulder, CO USA 80309

[5]Present address: Department of Cancer Immunology and Virology, Dana-Farber Cancer Institute, Boston, MA, USA 02115

*** Correspondence:**

yir@northwestern.edu

Figure S1

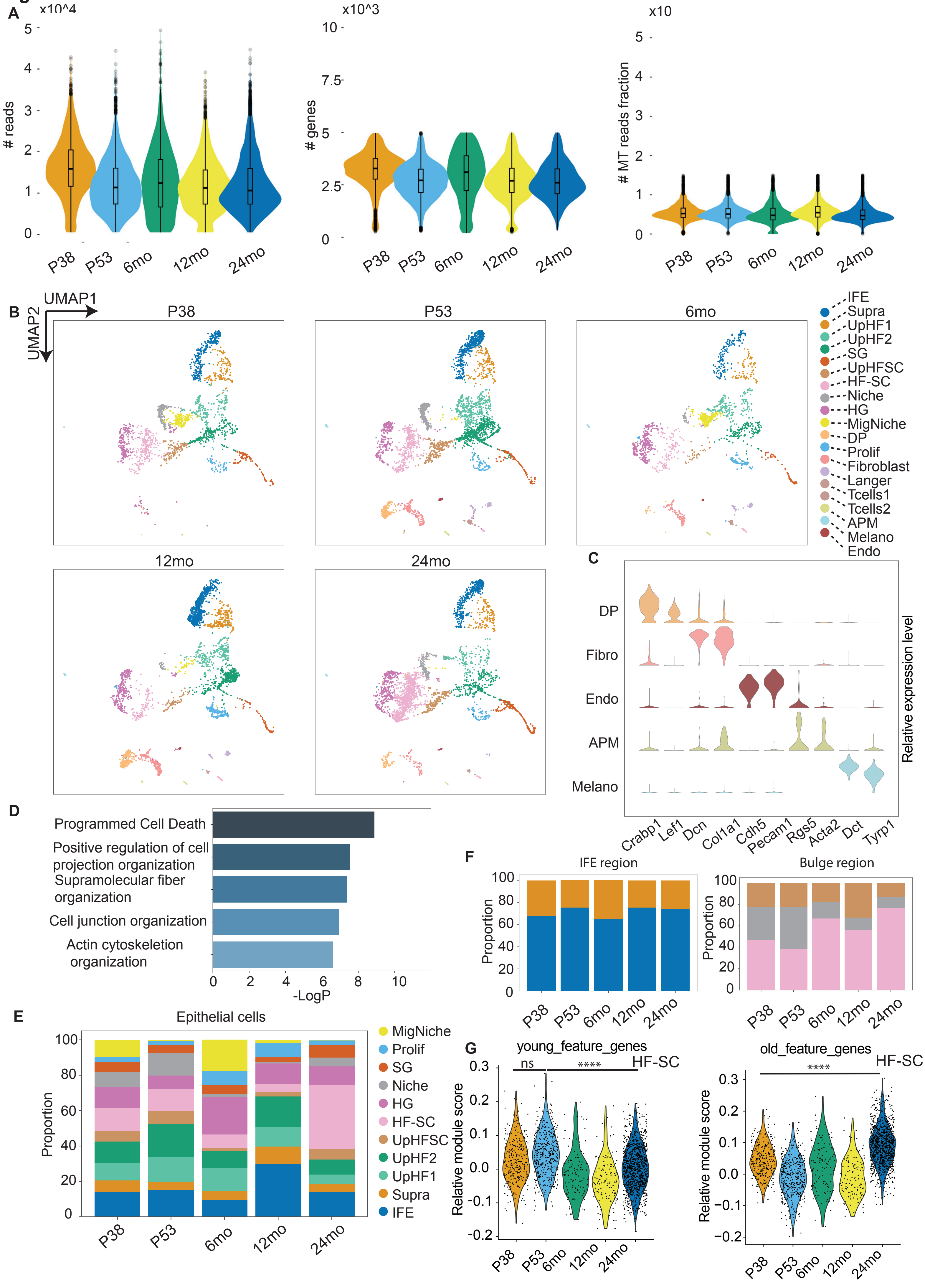
A
x10^4
reads
P38
P53
6mo
12mo
24mo
x10^3
genes
x10
MT reads fraction
B
UMAP1
UMAP2
P38
P53
6mo
12mo
24mo
IFE
Supra
UpHF1
UpHF2
SG
UpHFSC
HF-SC
Niche
HG
MigNiche
DP
Prolif
Fibroblast
Langer
Tcells1
Tcells2
APM
Melano
Endo
C
DP
Fibro
Endo
APM
Melano
Relative expression level
Crabp1
Lef1
Dcn
Col1a1
Cdh5
Pecam1
Rgs5
Acta2
Dct
Tyrp1
D
Programmed Cell Death
Positive regulation of cell projection organization
Supramolecular fiber organization
Cell junction organization
Actin cytoskeletion organization
-LogP
E
Epithelial cells
Proportion
MigNiche
Prolif
SG
Niche
HG
HF-SC
UpHFSC
UpHF2
UpHF1
Supra
IFE
F
IFE region
Bulge region
Proportion
G
young_feature_genes
old_feature_genes
ns
****
HF-SC
Relative module score

Figure S1. Quality control and clustering of scRNAseq data.

(A). Violin plots of number of reads, number of genes and mitochondrial fractions per sample. **(B).** UMAP clustering and population identification of individual sample. Each cell population was color coded and presented in all samples. **(C).** Marker genes violin plot of DP, Fibro, Endo, APM and Melano cells. **(D).** Highly enriched GO terms of upregulated genes in migNiche cells compared to Niche cells. **(E-F).** Compositional analysis of cell population proportions. IFE region and bulge region only contain anatomically adjacent populations. **(G).** Violin plots of young and old feature genes in HF-SCs. MT, mitochondrial; IFE, interfollicular epidermal basal cells; Supra, suprabasal cells; UpHF1/2, differentiated hair follicle cells in the upper portion; SG, sebaceous gland; UpHFSC, Lgr6+ HF-SCs; HF-SC, hair follicle stem cells; Niche, inner layer niche cells; HG, hair germ; MigNiche, migratory niche cells; DP, dermal papillae; Prolif, proliferating cells; Fibroblast, Fibroblast cells; Langer, Langerhan cells; Tcells1/2, T cells; APM, arrector pili muscle; Melano, melanocytes; Endo, endothelial cells.

Figure S2

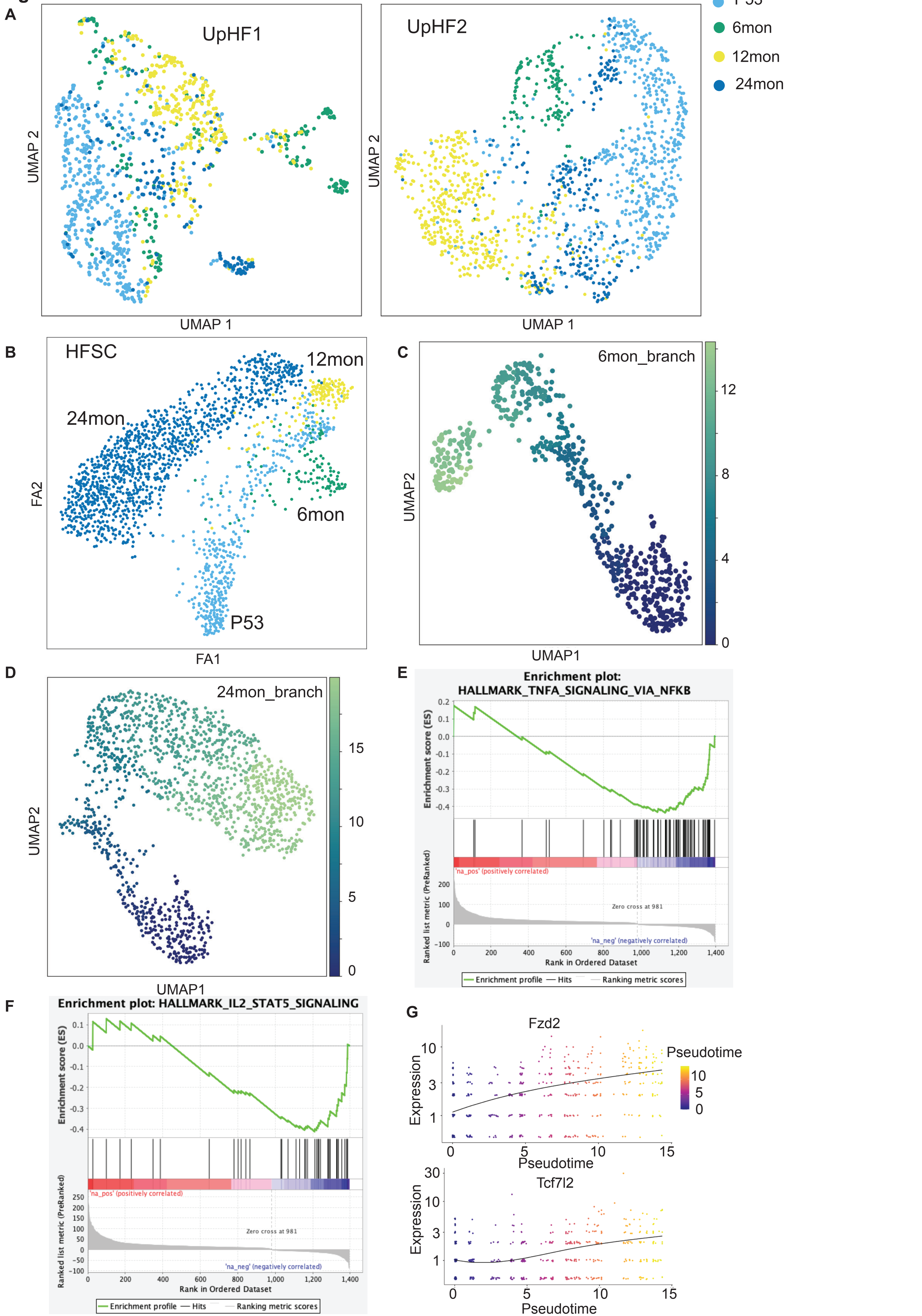
A
UpHF1
UpHF2
UMAP 1
UMAP 2
P53
6mon
12mon
24mon
B
HFSC
12mon
24mon
6mon
P53
FA1
FA2
C
6mon_branch
UMAP1
UMAP2
12
8
4
0
D
24mon_branch
UMAP1
UMAP2
15
10
5
0
E
Enrichment plot: HALLMARK_TNFA_SIGNALING_VIA_NFKB
Enrichment score (ES)
'na_pos' (positively correlated)
Zero cross at 981
'na_neg' (negatively correlated)
Ranked list metric (PreRanked)
Rank in Ordered Dataset
Enrichment profile
Hits
Ranking metric scores
F
Enrichment plot: HALLMARK_IL2_STAT5_SIGNALING
Enrichment score (ES)
'na_pos' (positively correlated)
Zero cross at 981
'na_neg' (negatively correlated)
Ranked list metric (PreRanked)
Rank in Ordered Dataset
Enrichment profile
Hits
Ranking metric scores
G
Fzd2
Tcf7l2
Expression
Pseudotime

Figure S2. Lineage specific aging trajectories of hair follicle lineages.

(A-B). UMAP visualization of differentiated hair follicle lineages UpHF1(left) and UpHF2(right), colored by samples. **(B).** Force directed graph visualization of aging HF-SCs, color-coded by samples. **(C, D).** Monocle3 pseudotime plot of 6mo branch**(C)** and 24mo branch**(D)** color-coded by pseudotime values. **(E, F).** Gene set enrichment analysis (GSEA) of 6mo and 24mo branch cells.

Figure S3

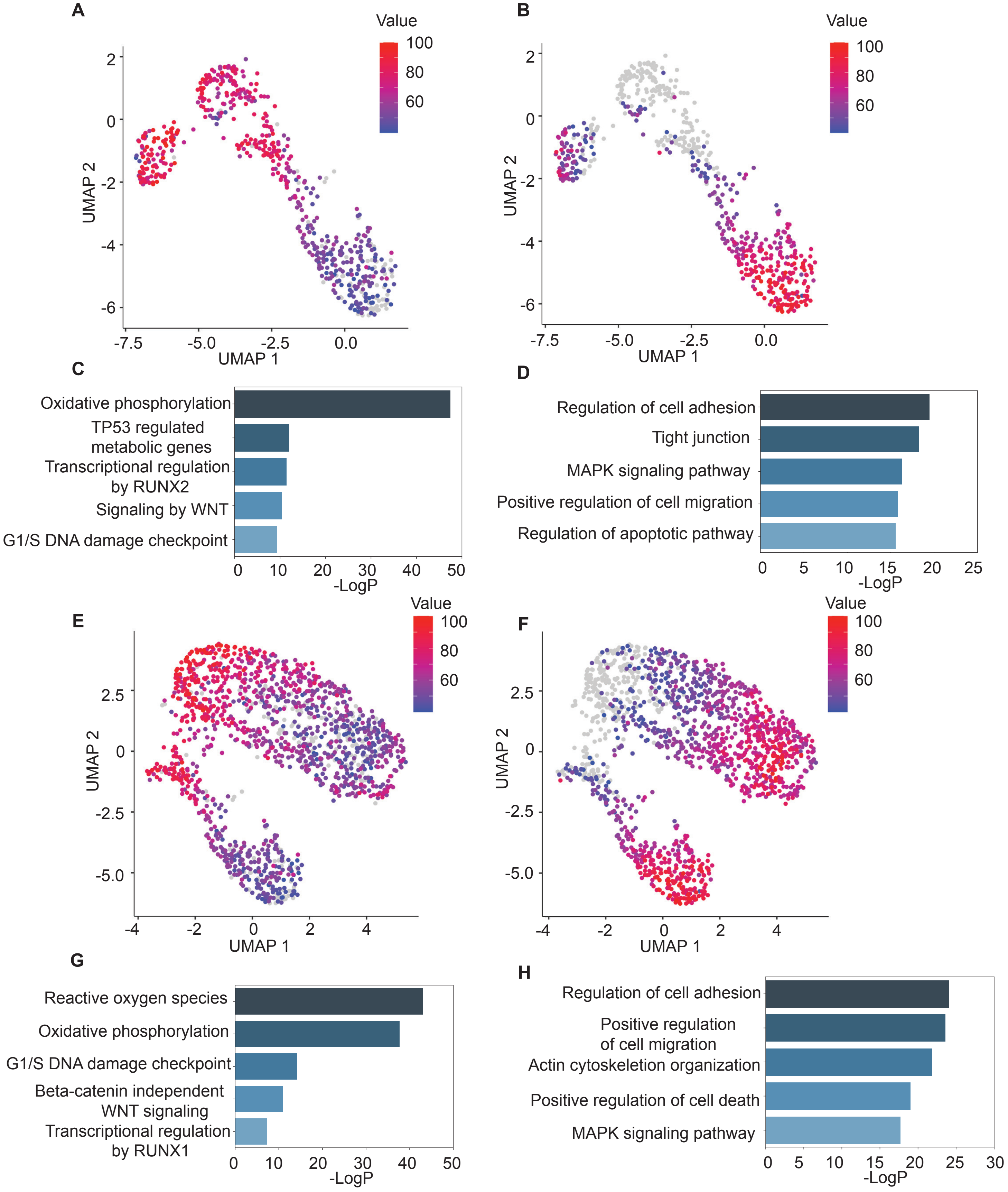
A
Value
100
80
60
UMAP 2
UMAP 1
B
C
Oxidative phosphorylation
TP53 regulated metabolic genes
Transcriptional regulation by RUNX2
Signaling by WNT
G1/S DNA damage checkpoint
-LogP
D
Regulation of cell adhesion
Tight junction
MAPK signaling pathway
Positive regulation of cell migration
Regulation of apoptotic pathway
E
F
G
Reactive oxygen species
Oxidative phosphorylation
G1/S DNA damage checkpoint
Beta-catenin independent WNT signaling
Transcriptional regulation by RUNX1
H
Regulation of cell adhesion
Positive regulation of cell migration
Actin cytoskeletion organization
Positive regulation of cell death
MAPK signaling pathway

Figure S3. Gene co-expression patterns along pseudotime trajectory. (A, B). Aggregated expression of all genes in two different co-expression modules along the 6mo branch. **(C, D).** GO term analysis of gene modules corresponding to **(A)** and **(B). (E, F).** Aggregated expression of all genes in different co-expression modules along the 24mo branch. **(G, H).** GO term analysis of gene modules corresponding to **(E)** and **(F)**.

Figure S4

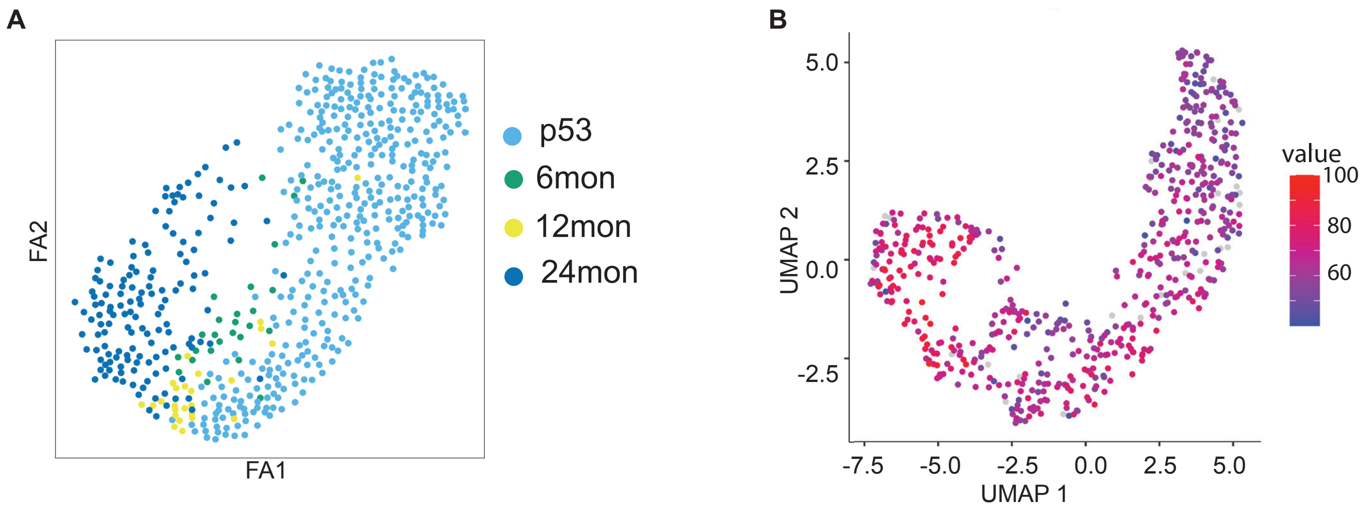
A
FA2
FA1
p53
6mon
12mon
24mon
B
UMAP 2
UMAP 1
5.0
2.5
0.0
-2.5
-7.5
-5.0
0.0
value
100
80
60

Figure S4. Niche cell dynamics during aging.

(A). Force directed graph visualization of Niche cells during aging, colored by samples. **(B).** Aggregated expression of all genes in Cluster3 gene modules along the Niche cells during aging.

Figure S5

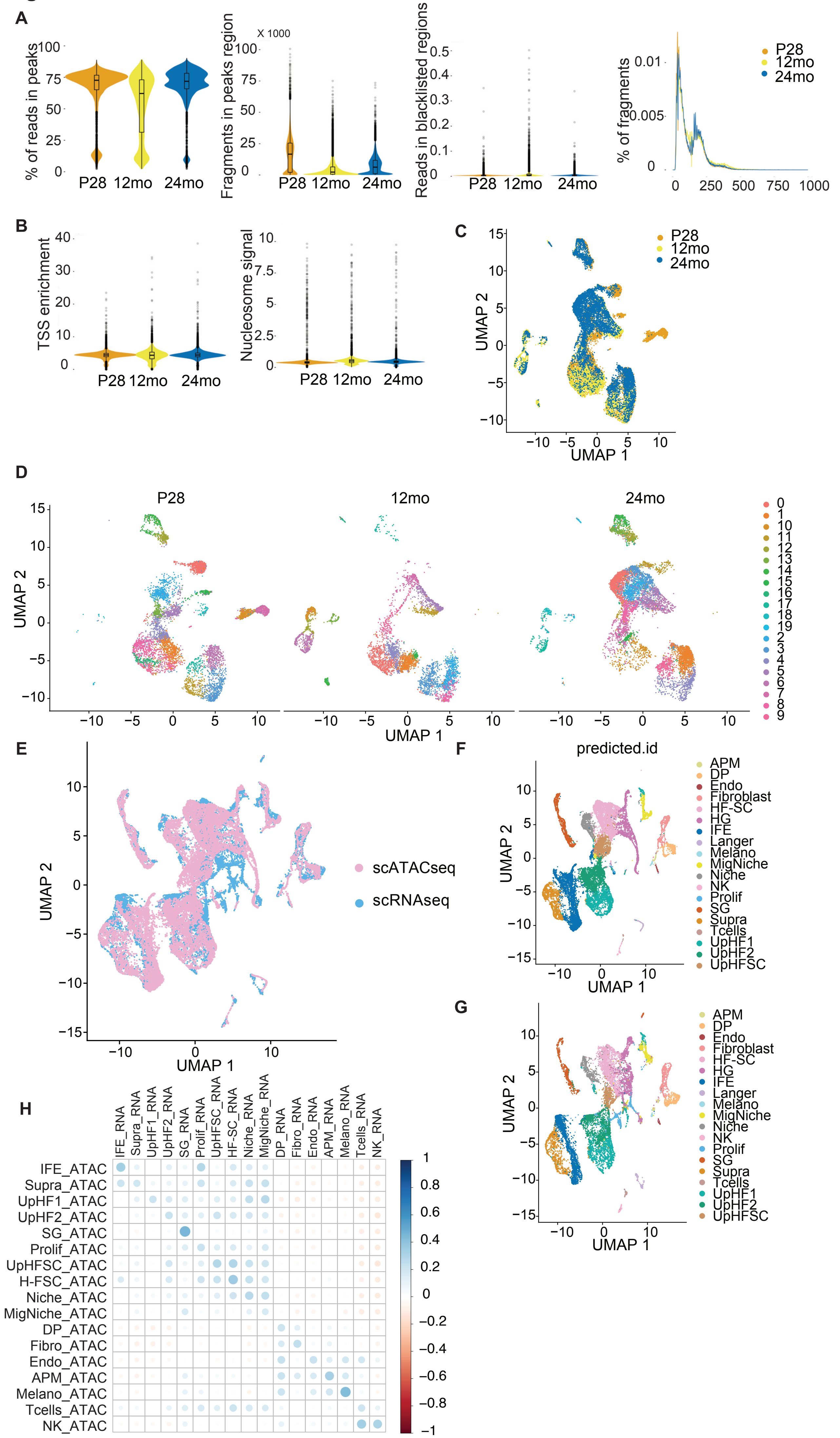

A
% of reads in peaks
P28 12mo 24mo
Fragments in peaks region
X 1000
Reads in blacklisted regions
% of fragments
P28
12mo
24mo
B
TSS enrichment
Nucleosome signal
C
UMAP 2
UMAP 1
D
P28
12mo
24mo
E
scATACseq
scRNAseq
F
predicted.id
APM
DP
Endo
Fibroblast
HF-SC
HG
IFE
Langer
Melano
MigNiche
Niche
NK
Prolif
SG
Supra
Tcells
UpHF1
UpHF2
UpHFSC
G
H
IFE_RNA
Supra_RNA
UpHF1_RNA
UpHF2_RNA
SG_RNA
Prolif_RNA
UpHFSC_RNA
HF-SC_RNA
Niche_RNA
MigNiche_RNA
DP_RNA
Fibro_RNA
Endo_RNA
APM_RNA
Melano_RNA
Tcells_RNA
NK_RNA
IFE_ATAC
Supra_ATAC
UpHF1_ATAC
UpHF2_ATAC
SG_ATAC
Prolif_ATAC
UpHFSC_ATAC
H-FSC_ATAC
Niche_ATAC
MigNiche_ATAC
DP_ATAC
Fibro_ATAC
Endo_ATAC
APM_ATAC
Melano_ATAC
Tcells_ATAC
NK_ATAC

Figure S5. Quality control and integration of scATACseq and scRNAseq data.

(A). Violin plot of percentage of reads in peaks, numbers of fragments in peaks and percentage of reads in blacklisted regions. Distribution of fragment length (right panel). **(B).** Violin plots of calculated TSS enrichment score and nucleosome signals among all samples. **(C).** UMAP visualization of integrated scATACseq samples. **(D).** UMAP plot of individual scATACseq sample. **(E).** Integration of scRNAseq and scATACseq samples using scRNAseq as reference. **(F, G).** UMAP visualization of coembeded scRNAseq(G) and scATACseq(F) samples, color-coded by cell populations. **(H).** Pearson correlation of all cell populations based on gene expression from scRNAseq and calculated gene activity score from scATACseq.

Fiugre S6

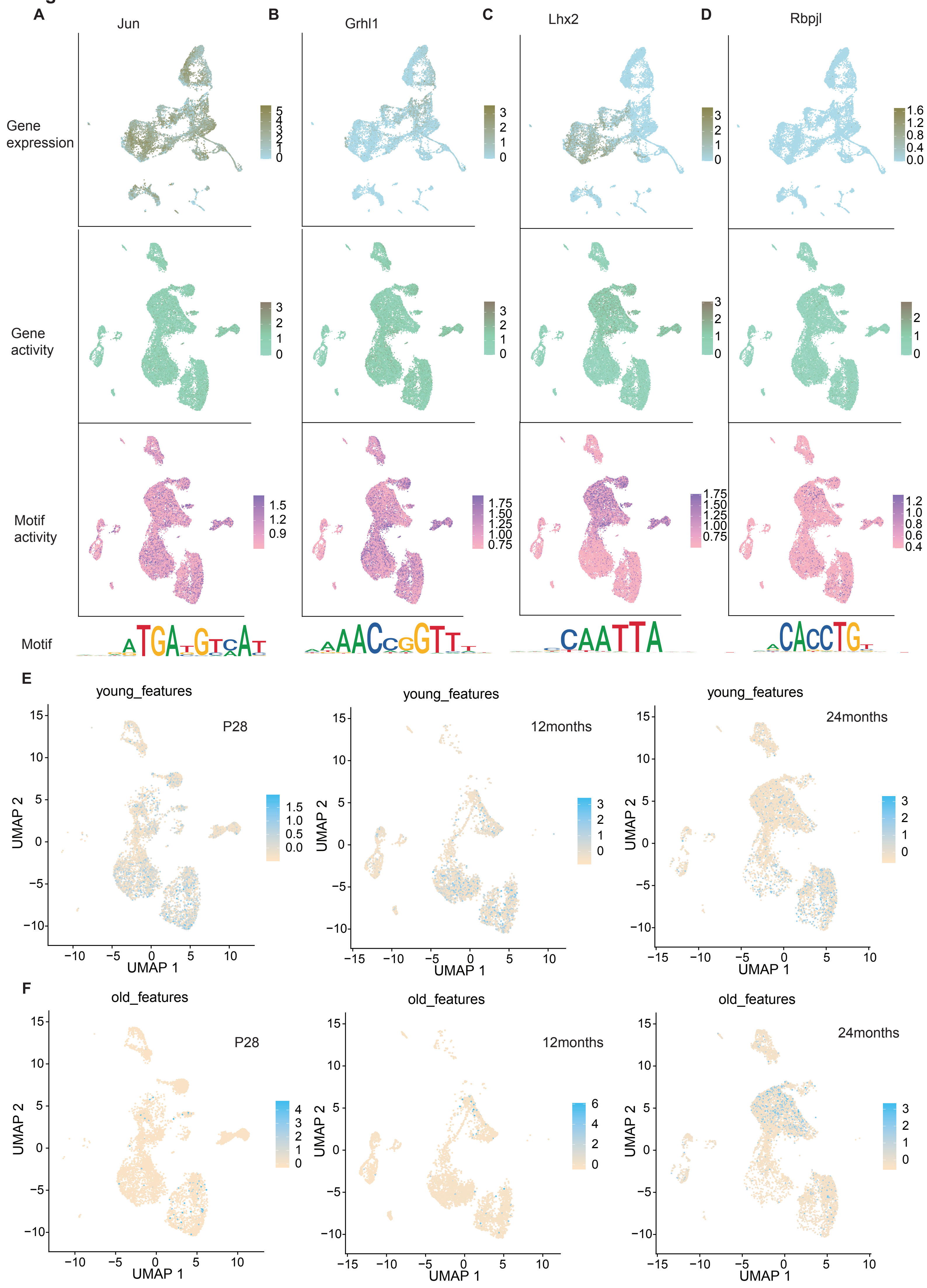
A
Jun
B
Grhl1
C
Lhx2
D
Rbpjl
Gene expression
Gene activity
Motif activity
Motif
E
young_features
P28
12months
24months
F
old_features
UMAP 1
UMAP 2

Figure S6. Open chromatin dynamics of hair follicle lineages during aging.

(A-D). Gene expression inferred by gene activity, motif activities and motifs plot of different transcription factors. **(E, F).** Feature plot of young and old open chromatin regions in individual samples.

Table S1 Mouse and sample information

Table S2 Differential genes in migNiche vs Niche

Table S3 Young and Old feature genes detected in RNAseq

Table S4 Differential genes of HF-SCs in 6mo vs 24mo branch

Table S5 gene modules detected in 6mo branch

Table S6 gene modules detected in 24mo branch

Table S7 Young and Old feature regions detected in ATACseq
